# The evolution and expression of stomatal regulators in C3 and C4 crops: Implications on the divergent drought tolerance

**DOI:** 10.3389/fpls.2023.1100838

**Published:** 2023-02-01

**Authors:** Zhuojun Song, Le Wang, May Lee, Gen Hua Yue

**Affiliations:** ^1^ Molecular Population Genetics and Breeding Group, Temasek Life Sciences Laboratory, 1 Research Link, National University of Singapore, Singapore, Singapore; ^2^ Department of Biological Sciences, National University of Singapore, Singapore, Singapore

**Keywords:** drought, tolerance, C3, C4, stomata, *SPEECHLESS*

## Abstract

Drought stress is a major environmental hazard. Stomatal development is highly responsive to abiotic stress and has been used as a cellular marker for drought-tolerant crop selection. C3 and C4 crops have evolved into different photosynthetic systems and physiological responses to water deficits. The genome sequences of maize, sorghum, and sugarcane make it possible to explore the association of the stomatal response to drought stress with the evolution of the key stomatal regulators. In this study, phylogenic analysis, gene expression analysis and stomatal assay under drought stress were used to investigate the drought tolerance of C3 and C4 plants. Our data shows that C3 and C4 plants exhibit different drought responses at the cellular level. Drought represses the growth and stomatal development of C3 crops but has little effect on that of C4 plants. In addition, stomatal development is unresponsive to drought in drought-tolerant C3 crops but is repressed in drought-tolerant C4 plants. The different developmental responses to drought in C3 and C4 plants might be associated with the divergent expression of their *SPEECHLESS* genes. In particular, C4 crops have evolved to generate multiple *SPEECHLESS* homologs with different genetic structure and expression levels. Our research provides not only molecular evidence that supports the evolutionary history of C4 from C3 plants but also a possible molecular model that controls the cellular response to abiotic stress in C3 and C4 crops.

## Introduction

Drought stress is the most critical environmental threat to global food security. The loss in crop yield caused by drought stress is larger than all biologically caused losses combined (Gupta et al., 2020). To adapt to water scarcity in soil, plants have evolved strategies to prevent water loss and maintain their key biological processes (Basu et al., 2016). During drought stress, roots display hydro-morphological changes by adjusting the lateral root emergence to soil with higher water content (Dinneny, 2019). This process is mediated by the EXOCYST SUBUNIT EXO70 FAMILY PROTEIN A3 (EXO70A3) *via* regulating the homeostasis of the auxin efflux carrier PINFORMED 4 (PIN4) in root tips (Ogura et al., 2019).

Stomata are valve-like openings found in the plant epidermis. The regulation of stomatal development and movement is important for plants to defend against dehydration (Lee and Bergmann, 2019). Stomatal lineage transitions and cell divisions are fine-tuned by three basic helix-loop-helix (bHLH) transcription factors: SPEECHLESS (SPCH) (Macalister et al., 2007), MUTE (Pillitteri et al., 2007) and FAMA (Ohashi-Ito and Bergmann, 2006) and their heterodimer SCREAM1 (Kanaoka et al., 2008). Among them, the expression and regulation of SPCH are highly responsive to the environment, which leads to the stomatal developmental response (Lee and Bergmann, 2019). High temperature represses the expression of *SPCH* by regulating the expression of PHYTOCHROME-INTERACTING FACTOR 4 (PIF4) at the transcriptional level (Lau et al., 2018). In *Arabidopsis*, drought induces the activity of the mitogen-activated protein kinase (MAPK) cascade to destabilize the expression of SPCH at the protein level, resulting in a reduction of stomatal density (Kumari et al., 2014). This has been used in the bioengineering of drought-tolerant crops. The overexpression of the extracellular secreted peptide EPIDERMAL PATTERNING FACTOR 2 (EPF2) enhances the drought tolerance of rice by activating the MAPK cascade (Hepworth et al., 2015). A recent study showed that SnRK2 kinases of the ABA signaling pathway directly phosphorylated SPCH in a drought-dependent manner, resulting in the change of stomatal production in response to drought stress (Yang et al., 2022).

Plants have evolved specific adaptation mechanisms to survive short- and long-term drought stresses. Hormones, microRNAs and other transcriptional factors are crosslinked with stomatal plasticity in response to abiotic stresses (Han et al., 2021). Drought stimulates the accumulation of the plant hormone abscisic acid (ABA), which mediates the signal crosstalk with other pathways, such as BRASSINOSTEROIDS pathway during drought stress (Song et al., 2016). The activation of ABA-responding genes, such as *SNF1-RELATED KINASE 2* (*SnRK2.2*), represses the stomatal development and induces stomatal closure (Chater et al., 2014). miR156 mediates stomata behavior under drought stress *via* ABA-dependent accumulation of strigolactones (Visentin et al., 2020). Stomatal movement is also required for drought tolerance and pathogen defense, which are mainly regulated by reactive oxygen species (ROS) and ABA-signaling (Qi et al., 2018). Many transcriptional factors are involved in the signaling pathways. Under a short-term or initial stage of drought stress, plants activate MYB60 expression and promote root growth to intake more water. By contrast, a long-term or severe drought stress repressed the MYB60 expression, resulting in root growth inhibition and stomatal closure to prevent water loss (Oh et al., 2011). Other key TFs, including AP2/ERF, bZIP, WRKY, YABBY and NAC, are also involved in stomata-dependent drought tolerance (Hussain et al., 2021).

C4 species are essential to the tropical ecosystems (Still et al., 2003) and important agricultural crops (e.g. maize, sugarcane, sorghum). C4 photosynthesis is a marvelous functional evolution for plants. It increases the photosynthetic efficiency at high temperatures, drought and low CO_2_ levels through separating the carbon fixation from Ribulose-1,5-bisphosphate carboxylase-oxygenase (RuBisCO) and concentrating CO_2_ around RuBisCO (Sharwood et al., 2016). Dicot C4 plants originated in arid areas, indicating the effects of heat, drought, and salinity as important forces in promoting C4 evolution (Sage, 2004). C4 plants exhibit lower stomatal conductance, which is correlated with their higher water and nitrogen use efficiency compared to C3 species (Taylor et al., 2010). Comparative genomic studies reveal that the evolution of C4 plants depends on whole-genome and individual gene duplication (Wang et al., 2009; Van Den Bergh et al., 2014). The C4 gene homologs may have different adaptive evolution and duplicability (Wang et al., 2009). Interestingly, the rice phosphoenolpyruvate carboxylase gene undergoes rapid evolution and shows C4-like pattern (Wang et al., 2009).

Stomatal development and their response to osmotic stresses are largely studied in C3 plants. Some tropical-grown crops, such as oil palm, exhibit higher salt tolerance due to the divergent regulation of the expression of *SPCH* (Song et al., 2022). However, the molecular mechanism of how stomata respond to drought stress in C4 plants and the evolutionary divergence of the stomatal developmental regulators are largely unknown. In this study, by using a comparative genomic approach combined with physiological and molecular analysis, we investigated the stomatal response to drought, and the divergent genetic structure and expression of stomatal genes in major C3 and C4 crops. Our findings will facilitate the understanding of the molecular basis of the different drought tolerance and response in C3 and C4 plants.

## Material and methods

### Plant growth and drought challenge

*Arabidopsis* col-0 seeds were transferred onto sterilized soil and were kept in a darkroom at 4°C for 3-days. Seedings were germinated and grown in a plant growth chamber at 22°C with 60% relative humidity under long-day conditions (16 h light/8 h dark) at a light intensity of 100 μmol m^–2^ s^–1^. For the drought assay, 7 dpg (days post germination) seedlings were either continually watered (control group) or not watered (drought group) for another 7 days (14 dpg with 7 das (days after stress)). Cotyledons from over 20 seedlings were used for stomatal analysis.

Two-year-old oil palm (*Elaeis guineensis*) seedlings, one-year-old sugarcane (*saccharum spontaneum*) and sorghum (*Sorghum bicolor*) plants were grown in a greenhouse with natural tropical environment. Samples from the control group were watered daily while samples from the drought stress group were not watered for 14 days. Over 20 fresh rosette leaves from more than 4 seedlings of each group were used for stomatal analysis. Drought-tolerant oil palms were screened in our previous study (Wang et al., 2020). Drought-tolerant sugarcanes were screened during the drought challenge in the green house.

The seeds of japonica rice (*Oryza sativa*) were geminated and grown in a petri dish with 20 mL water in the plant growth chamber with a light/dark cycle of 12 h 28°C/12 h 26°C. At 14 dpg, seedlings were transferred into sterilized soil. For drought and stomatal assay, 14 dpg rice seedlings were transferred into the pots with either wet soil (control group) or dry soil (drought group) for another 7 days (21 dpg with 7 das). 21 dpg seedlings were used for stomatal analysis. Over 20 first leaves from more than 20 rice seedlings of each group were used for analysis.

### Stomatal assay

Freshly collected leaves were cleared in a 7:1 ethanol:acetic acid buffer overnight and mounted in a 8:2:1 chloral hydrate: water: glycerol clearing buffer for 24 hours. The abaxial leaf epidermis of the leaf slides was captured at 20 × on a Leica DM2500 microscope with a differential contrast interference (DIC) channel. Two images at either 0.25 mm^2^ or 0.0625 mm^2^ were captured per leaf from the central regions. The stomatal density was counted by built-in tool the ‘cell counter’ in ImageJ (NIH, USA).

### Constructing phylogenetic trees and analyzing protein structure

The current annotation of the protein sequences of sugarcane is still poor. To identify as many homologs of stomatal regulators in sugarcane as possible, the known protein sequences of the three stomatal regulating genes SPCH, MUTE and FAMA of some C3 and C4 plants were used as probe for tBLASTn (McGinnis and Madden, 2004) with a minimum p-value of 1e^-30^. These C3 and C4 plants are *Arabidopsis* (*At*, *Arabidopsis thaliana*), Oil palm (*Eg*, *Elaeis guineensis*), potato (*St, Solanum tuberosum*), grape (*Vv, Vitis vinifera*), japonica rice (*Os.j, Oryza sativa*), soybean (*Gm, Glycine max*), tomato (*Sl, Solanum lycopersicum*), maize (*Zm, Zea mays*), sugarcane (*Ss, saccharum spontaneum*) and sorghum (*Sb, Sorghum bicolor*). After which, the homolog candidates were filtered and annotated by BLASTn (McGinnis and Madden, 2004) against the standard nucleotide database. The filtered CDS sequences of the homologs were aligned with the sugarcane proteome reference (Zhang et al., 2018). The validated protein sequences were aligned using the built-in ClustalW (Hung and Weng, 2016) in MEGA-X software (Kumar et al., 2018) by default settings. The protein FLOWERING LOCUS T (FT) (Turck et al., 2008) was used as the outgroup reference. The accession IDs of the protein sequences were listed in **Supplementary Table 1**. A phylogenetic tree was constructed by using the maximum likelihood method (Strimmer and Von Haeseler, 1996) with 100 bootstraps *via* MEGA-X software (Kumar et al., 2018). The protein structure was reconstructed by the ColabFold software (Mirdita et al., 2022) based on AlphaFold (Jumper et al., 2021) using the default settings.

### RNA extraction, cDNA synthesis and Q-PCR

Total RNA from leaves was extracted using the RNeasy Plant Mini Kit (Qiagen, Germany). RNA quality and quantity were assessed using a previously described method (Song et al., 2022). cDNA was synthesized using M-MLV reverse transcriptase (Promega, USA) following the manufacturer’s protocol. RT-qPCR was performed in a CFX96 touch deep well real time PCR System (Bio-Rad, USA) with the program in a previous study (Liu et al., 2020). RT-qPCR was used to examine the expression of the stomatal regulating genes SPCH, MUTE and FAMA from *Saccharum spontaneum*, *Sorghum bicolor* and *Oryza sativa* for qPCR. β-TUBLIN genes were used as an internal control to normalize the relative expression of genes. A standard cycling program was used for qPCR: initial denaturation at 94°C for 2 min, followed by 40x cycles of denaturation at 94°C for 15s and annealing at 65°C for 1 min. The primers used for Q-PCR are listed in **Supplementary Table 2**.

## Results

### Stomatal development and response to drought stress in C3 and C4 plants

**Figure 1:**
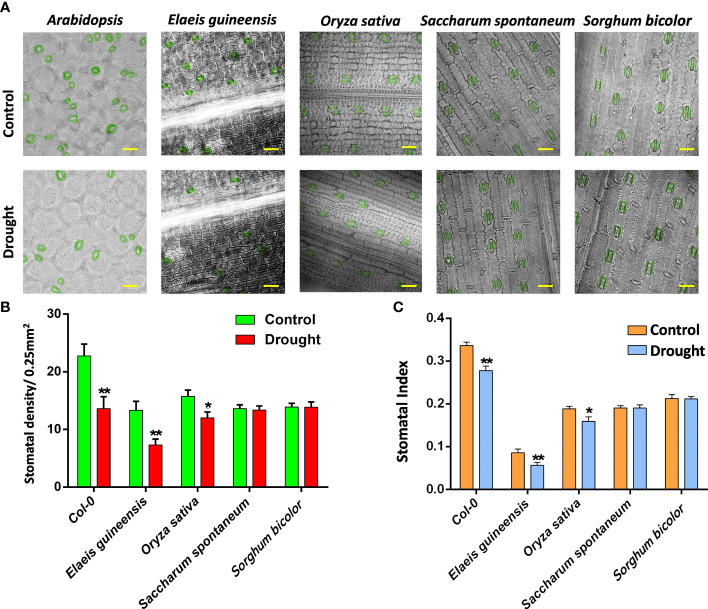
**(A)** The abaxial stomatal pattern of 14 dpg *Arabidopsis*, two-year-old *Elaeis guineesis*, 21 dpg *Oryza sativa*, one-year-old *Saccharum spontaneum* and one-year-old *Sorghum bicolor* under control and drought stress. Bar =25 *um*. **(B)** The stomatal density of samples from **(A)**. **(C)** The stomatal index of samples from **(A)**. The values are mean ± SEM; n ≥ 20. One-way ANOVA with *post hoc* Tukey HSD; *, p <0.05; **, p<0.01.

As monocots, C3 plants, including oil palm and rice, showed a comparable stomatal density with C4 plants, including sugarcane and sorghum (**Figures 1A, B**), suggesting the similar stomatal pattern and production of monocots. However, rice showed a much lower stomatal index compared to the others due to its relatively higher number of pavement cells (**Figures 1B, C**). After drought treatment, the stomatal density and index of C3 plants, including *Arabidopsis*, oil palm and rice, were reduced, showing that drought represses the stomatal development of C3 plants. However, the stomatal development of C4 plants including sugarcane and sorghum were not affected by drought stress (**Figure 1B**). The stomatal index was measured to determine whether the possible reduction of the total epidermal cells caused the reduction in stomatal density. All the stomatal indexes of C3 and C4 plant groups showed the same trend with their stomatal densities after drought treatment (**Figures 1B, C**). The stomatal indexes of C3 plants were reduced whereas those of C4 plants were unchanged (**Figure 1C**), indicating that drought repressed the stomatal development of C3 plants but not C4 plants.

### Stomatal response to drought stress in drought-tolerant oil palm and sugarcane

**Figure 2:**
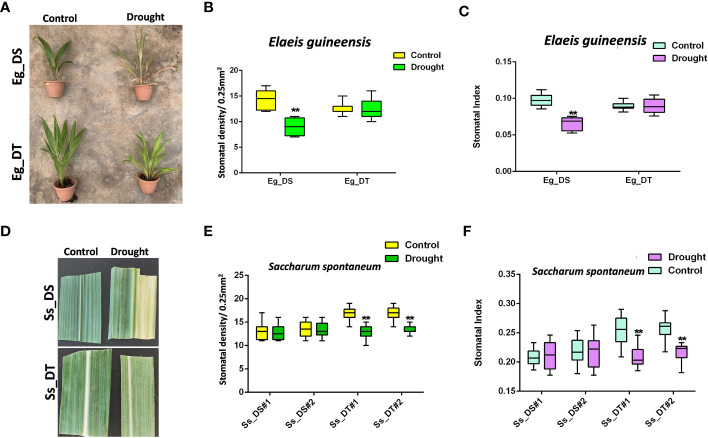
**(A)** The drought assay of drought-susceptible (Eg_DS) and drought-tolerant (Eg_DT) oil palms. Two-year-old oil palms were either continually watered or not watered for 14 days; n ≥ 4. **(B)** The stomatal density of samples from **(A)**. **(C)** The stomatal index of samples from **(A)**. **(D)** The drought assay of drought-susceptible (Ss_DS) and drought-tolerant (Ss_DT) sugarcanes. **(E)** The stomatal density of samples from **(D)**. **(F)** The stomatal index of samples from **(D)**. One-year-old sugarcanes were either continually watered or not watered for 21 days; n ≥ 2. The values are mean ± SEM; n ≥ 20. One-way ANOVA with *post hoc* Tukey HSD; **, p<0.01.

The stomatal response to drought stress of drought-tolerant C3 and C4 plants, compared to their drought-susceptible control, was tested using oil palm and sugarcane (**Figures 2A, D**). After drought treatment, leaf yellowing, leaf top necrosis, and other morphological changes were observed in both the drought-susceptible oil palm and sugarcane, although sugarcane could endure longer periods without water before its growth was affected (**Figures 2A, D**). However, the growth of drought-tolerant C3 and C4 crops was not affected (**Figures 2A, D**). The stomatal development of drought-tolerant oil palm and drought-susceptible sugarcane was unresponsive to drought stress (**Figures 2B, C, E, F**), which showed that the drought-susceptible sugarcane naturally has a higher tolerance to drought compared to C3 crops. Interestingly, the drought-tolerant sugarcane showed reduced stomatal development similar to a drought-susceptible C3 crop. (**Figures 2E, F**). In addition, drought-tolerant oil palm showed a lower stomatal density compared to the drought-susceptible oil palm (**Figures 2B, C**) whereas the stomatal density of drought-tolerant sugarcane was higher than the drought-susceptible sugarcane. After a 21 dpg (**Figure 2D**) intense drought treatment, the stomatal density was reduced to the same level of the susceptible sugarcane (**Figures 2E, F**). These data suggest the different physiological responses and molecular mechanisms of C3 and C4 crops under drought stress.

### Genetic divergence of stomatal regulators SPCH, MUTE and FAMA in C3 and C4 plants

**Figure 3:**
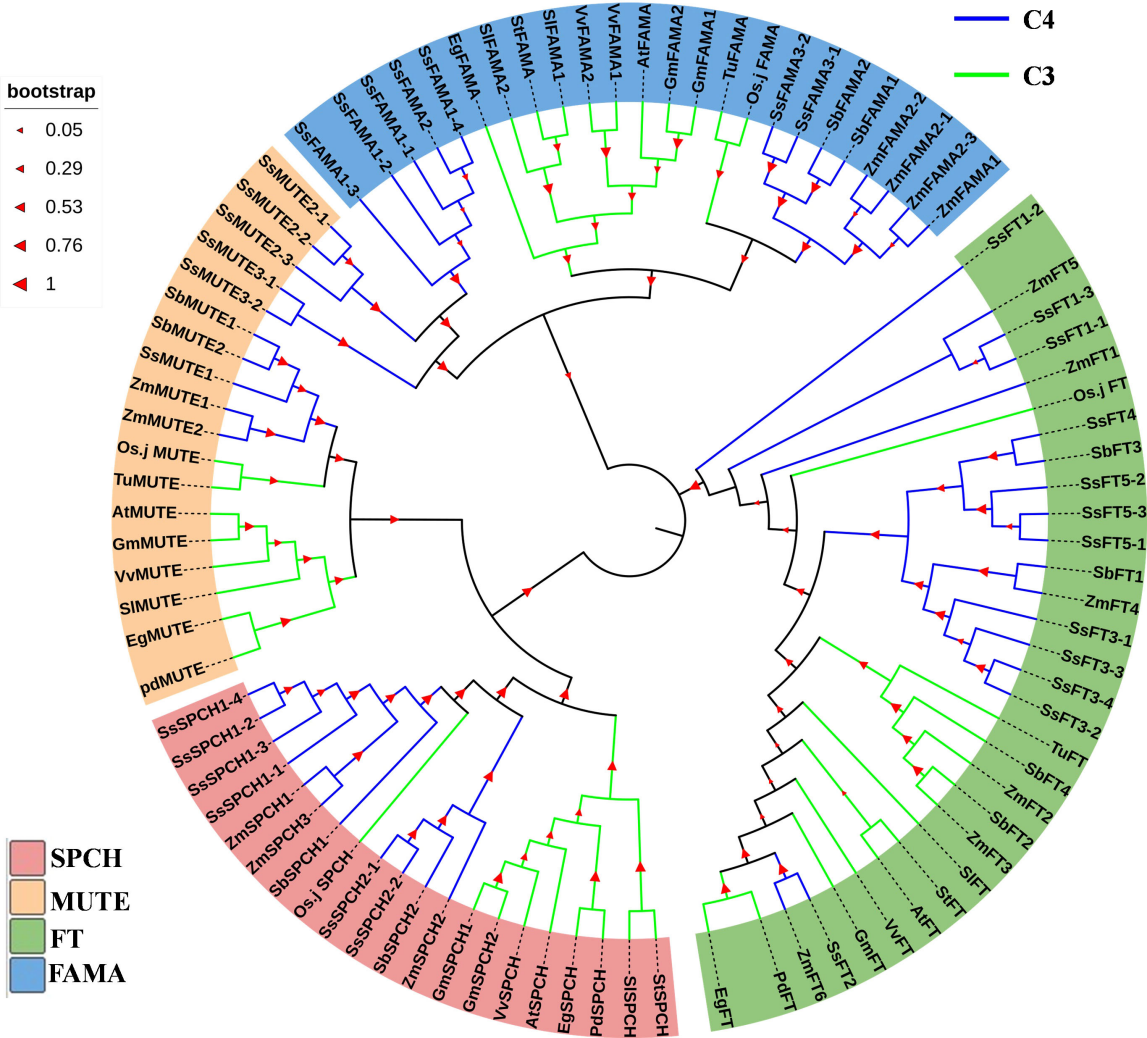
The phylogenic analysis of the protein of SPCH and its two homolog transcription factors MUTE and FAMA in *Arabidopsis* (At), *Elaeis guineensis* (Eg), *Phoenix dactylifera* (Pd), *Oryza Sativa.japonica* (Osj), *Gycine max* (Gm), *Triticum urartu* (Tu), *Solanum lycopersicum* (Sl), *Solanum tuberosum* (St), *Vitis vinifera *(Vv), *Zea mays* (Zm), *Sorghum bicolor* (Sb), *Saccharum spontaneum* (Ss). The protein FLOWERING LOCUS T (FT) of above species was used as the outgroup. Bootstrap =500; The percentage of replicate trees in which the associated taxa clustered together in the bootstrap test (500 replicates) is shown on the branches.

In general, genetic divergence of SPCH, MUTE and FAMA was found between C3 and C4 plants (**Figure 3**). Only a few C3 crops had homologs of the above three proteins (**Figure 3**). In contrast, there were 2–3 groups of homologs for each of SPCH, MUTE, and FAMA in C4 plants (**Figure 3** and **Supplemental Table S1**). Each group contained several homologs with highly conserved genetic similarity. The homologs were on either homologous or different chromosomes (**Figure 3** and **Supplemental Table S1**). The MUTE and FAMA proteins of C3 and C4 plants were highly differentiated (**Figure 3**). However, C4 plants exhibited two genetic patterns of MUTE and FAMA (**Figure 3**). SsFT2 and ZmFT6 showed typical C3-like pattern, SsSPCH2-1/2-2, SbSPCH2 and ZmSPCH2 were closer to the C3 SPCH group (**Figure 3**). Interestingly, among the C3 plants used for our phytogenic analysis, rice showed the closest genetic relationship with C4 plants (**Figure 3**). The FT and SPCH of rice showed an intermediate genetic pattern between not only C3 and C4, but also a C3-like C4 group and a C4-pattern C4 group (**Figure 3**). These data mirrored the different pace of evolution of SPCH and FT proteins from C3 to C4 plants.

### Divergent expression of *SPCH* in response to drought stress

**Figure 4:**
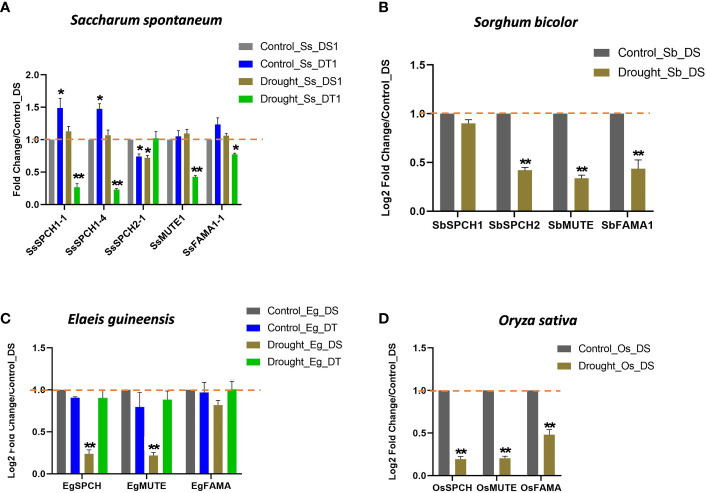
The relative expression levels of *SPCH*, *MUTE* and FAMA in **(A)** the drought-susceptible and tolerant *Saccharum spontaneum*; **(B)** *Sorghum bicolor;* **(C)** the drought-susceptible and tolerant *Elaeis guineensis* and **(D)** *Oryza sativa*. Triplicates were included in the Q-PCR, the expression level of drought-susceptible plants under control was standardized to 1. The values are mean ± SEM; n ≥ 3. One-way ANOVA with *post hoc* Tukey HSD; *, p <0.05; **, p<0.01.

The expressions of *SPCH*, *MUTE*, and *FAMA* in oil palm and rice were downregulated in the drought-susceptible plants under drought stress (**Figures 4C, D**). However, the expressions of these three genes were basically unchanged in the drought-tolerant oil palm under drought stress (**Figure 4C**), suggesting the importance of the SPCH signalling pathway in drought tolerance.

The expressions of three sugarcane *SPCH*-*SsSPCH1-1*, *SsSPCH1-4*, *SsSPCH2-1* (C3-like) and two sorghum *SPCH*- *SbSPCH1*, Sb*SPCH2* (C3-like) were analysed (**Figures 4A, B**). Interestingly, C4 and C3-like *SPCH* showed different expressions in response to drought (**Figures 4A, B**). Drought stress had few effects on the expression of C4 *SPCH* including *SsSPCH1-1*, SsSPCH1*-4* and *SbSPCH1* in drought-susceptible sugarcane and sorghum but repressed the expressions of C3-like *SsSPCH1-3* and *SbSPCH2* (**Figures 4A, B**). By contrast, drought strongly repressed the expressions of *SsSPCH1* and *SsSPCH2* but not *SsSPCH1-3* in drought-tolerant sugarcane (**Figures 4A, B**). Taken together, these data and the stomatal response to drought suggest that the regulation of C4 *SPCH* plays a dominant role in regulating the stomatal response of C4 plants to drought stress

### The structural difference of SPCH in C3 and C4 plants

**Figure 5:**
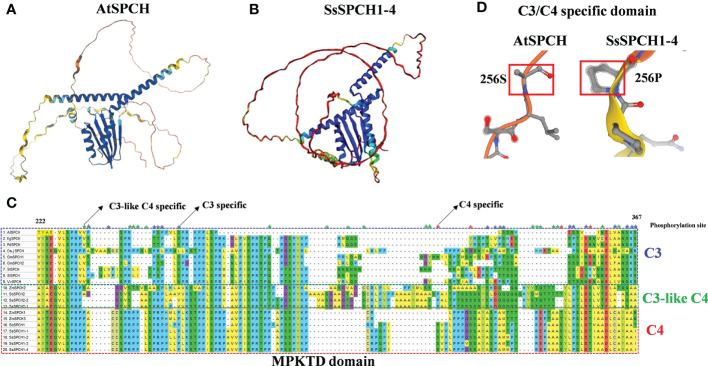
The 3D protein structure of **(A)** *Arabidopsis* SPCH and **(B)** Sugarcane SPCH1-4; **(C)** The alignment of MPKTD domains of C3 and C4 plants. The blue, red and green frame indicate the MPKTD sequence of C3, C4 and C3-like C4 SPCH. The blue, red and green asterisks indicate the C3-, C4, and C3-like C4- specific phosphorylation sites (S/T) within the MPKTD domain; **(D)** The C3/C4 specific S/T site (256) of AtSPCH and SssSPCH1-4.

The protein sequence and 3D protein structure of the C3 and C4 SPCH were analysed to determine the structural divergence of proteins, and their possible association with the phosphorylation of SPCH (**Figure 5**). Although SPCH of *Arabidopsis* and sugarcane showed a highly conserved helix-loop-helix (bHLH) domain (**Supplemental Figure S1**) and a similar bHLH structure (**Figures 5A, B**, blue), the folding of the peptide and structures of other domains were largely different (**Figures 5A, B**). Specifically, the MAPK target domain (MPKTD) of C3 and C4 SPCH exhibited high diversity (**Figures 5C, D**), suggesting the different phosphorylation sensitivity of C3 and C4 SPCH to upstream MAPKs. In summary, 17 C3-specific and 4 C4-specific Serine/Threonine (S/T) phosphorylation sites were found within the MPKTD (**Figure 5C**). Interestingly, 18 C3-like C4-specific S/Ts were found (**Figure 5C**), suggesting the SPCH homologs in C3 and C4 plants may exhibit different expressions at both transcriptional and post-translational levels. C3- and C4- specific insert/deletion (InDel) polymorphisms were found in the MPKTD, which may also affect the function of SPCH. In addition, the upstream (N-Terminus) of SPCH in C3 and C4 crops also showed high polymorphism, including SNPs and InDels (**Supplemental Figure S1**). These data indicate that the functional domain and sequences of C3 and C4 SPCH protein have undergone different evolution, which may lead to different levels of phosphorylation *via* MPKs signalling pathways and the stomatal lineage cell transition

## Discussion

### Stomatal development of C3 and C4 plants in response to drought stress

Crops exhibit different drought tolerances. To ensure that each species underwent adequate drought stress, the plants were treated with different durations of drought according to previous studies (Aharoni et al., 2004; Hura et al., 2007; Quan et al., 2010; Jangpromma et al., 2012; Zhang et al., 2015; Wang et al., 2020). *Arabidopsis* and rice seedlings show lower drought tolerance, therefore they were not given water for seven days (Aharoni et al., 2004; Quan et al., 2010). Tropical C3 crops like oil palm show a relatively higher drought tolerance than other temperate climate-grown C3 crops, thus, a 14-day drought treatment was used for oil palm as previously described (Wang et al., 2020). C4 plants were naturally more tolerant to drought than C3 plants (Hura et al., 2007). Seven- to nine-day drought assays were used in previous studies of sugarcane (Jangpromma et al., 2012; Zhang et al., 2015). However, a 9-day drought treatment did not induce obvious growth repression in our sugarcane (data not shown). Thus, we extended the drought treatment to 14 days until leaf yellowing was found in some sugarcane.

The result showed that drought strongly repressed the stomatal development of C3 plants but did not affect that of C4 plants (**Figure 1**), suggesting that C4 plants exhibit higher drought tolerance at the cellular level. The stabilization of stomatal development under drought may be helpful for C4 plants to maintain their photosynthesis efficiency. In addition, as sibling species, sugarcane and sorghum exhibited almost the same stomatal developmental pattern and response to drought (**Figure 1**). By contrast, the stomatal developmental pattern varied among C3 plants (**Figure 1**).

We obtained the drought-tolerant seedlings of two tropical crops, oil palm (Wang et al., 2020) and sugarcane, *via* drought assays. The data revealed that the stomatal development of drought-tolerant oil palm was not affected by drought (**Figures 2B, C**), which was similar to drought-susceptible sugarcane (**Figures 2E, F**). This result is consistent with the results in other drought-tolerant C3 crops where the cell development and homeostasis of physiological processes are not affected by drought stress (Mehri et al., 2009; Yoo et al., 2010; Liu et al., 2012). Therefore, the maintenance of stomatal development is required for stabilizing the biological process of C3 plants during drought.

Although C4 plants show a relatively higher drought tolerance compared to C3 plants (Taylor et al., 2010), long periods of drought still affected the growth and development of sugarcane (**Figure 2D**). Nevertheless, the stomatal development was not affected by drought stress (**Figures 2E, F**). Interestingly, the stomatal development of drought-tolerant sugarcane was repressed by drought (**Figures 2E, F**). These data suggest that during the evolution of C4 plants, the stomatal developmental system may have evolved to be unresponsive to drought stress, which would facilitate gas exchange during highly efficient photosynthesis. However, only drought-tolerant C4 plants may further protect themselves from drought damage by reducing water through ‘sensing’ the drought signal *via* their stomata. To validate this hypothesis, it would be interesting to test the stomatal response to drought in more C4 species.

### SPCH homologs undergoes differential evolution in C4 plants

Although the sugarcane gnomes have been sequenced at the monoploid (Garsmeur et al., 2018) and polyploid levels (Zhang et al., 2018), the extreme complexity of the sugarcane genome makes it difficult to analyze and annotate the genome (Thirugnanasambandam et al., 2018). The modern sugarcane has a duplicated genome originating from *S. officinarum* and *S. spontaneum* (D’hont et al., 2008). We identified at least six SPCH homologs in the polyploid genome (Zhang et al., 2018) to investigate the effects of gene duplication on the function of stomatal regulators. Our result showed that MUTE and FAMA were specifically evolved between C3 and C4 plants (**Figure 3**). In contrast, SPCH homologs of C4 species exhibited C4 and C3-like patterns (**Figure 3**). These data indicate the divergent evolution of SPCH homologs in C4 crops. The varied chromosomal structure, the interspecific hybridization, and the diverse growth habitat may play a key role in the duplication and evolution of sugarcane homologs (Thirugnanasambandam et al., 2018). As a key regulator of the rapid response of stomata to the environment (Lau et al., 2018), sugarcane SPCH homologs may have originated from ancestors that underwent divergent selection (Thirugnanasambandam et al., 2018). However, the SPCH homologs of C4 plants in this study might not cover all the homologs due to the complexity of the C4 plant genomes. Improved genome assemblies of C4 crops in the future would be helpful to better understand the functional genetics of sugarcane in response to environmental stresses.

In our study, the genetic relationship of rice’s FT and SPCH are in between of the C4-type and C3-like FT & SPCH of C4 plants (**Figure 3**). Our study supports the previous study that some rice genes have rapidly evolved into C4-like pattern (Wang et al., 2009). These studies pave the way to bio-engineer C4 rice (Ermakova et al., 2020).

### Regulation of SPCH expression at transcriptional and post-translational level

As a master transcription factor, the transcription of *SPCH* is also regulated by other transcription factors under different environmental changes (Lau et al., 2014; Lau et al., 2018). Interestingly, the *SPCH* homologs showed different expressional changes in response to drought stress (**Figures 4A, B**). Expressions of *SsSPCH1-1*, *SsSPCH1-4* and *SbSPCH1* were unresponsive to drought in drought-susceptible C4 plants but were repressed in drought-tolerant C4 plants (**Figures 4A, B**), which explained their cellular response to drought stress (**Figure 2**). The pseudogenes are largely identified in the sugarcane genome (Monteiro-Vitorello et al., 2004). In our study, *SsSPCH2-1* and *SbSPCH2* showed a C3-like expression repressed by drought stress (**Figures 4A, B**), which was opposite to the stomatal response of C4 plants to drought (**Figures 2D-F**). It was hypothesized that the C3-like *SPCH* in C4 plants may either be non-functional pseudogenes or have other drought-independent functions. Further functional validations of the promoter activities of these homolog genes would be helpful to test which of these homologs are functional and which of them are pseudogenes. Despite the difficulty in testing the function of each *SPCH* gene in C4 plants, our research reveals the transcriptional response of key stomatal regulators in C4 crops under drought stress. The divergent expressions of C4 *SPCH* homologs are associated with the drought response of C4 plants at the cellular level.

In C3 plants, drought also represses SPCH expression at the protein level *via* the MPK3/6 signaling pathway (Lee and Bergmann, 2019) and other kinase-dependent pathways (Yang et al., 2022). Serine/Threonine residues are required for the binding of MAPKs and SPCH, resulting in the phosphorylation of SPCH (Lampard et al., 2008). Although the bHLH domains of SPCH were highly conserved in plants (**Supplemental Figure S1**), the folding structure and key MPKTD domains were largely different (**Figure 5**), which might lead to different binding affinity of SPCH to both upstream kinases and downstream target genes, resulting in the different expression of SPCH at post-translational level in response to drought stress. The bHLH domain is required for the brassinosteroid dependent stomatal formation (De Marcos et al., 2017). There are S/T phosphorylation sites located upstream of SPCH which are also required for its function in stomatal lineage cell transition (Davies and Bergmann, 2014). Although the bHLH domain is highly conserved across C3 and C4 plants, the N-terminus, C-terminus and other functional domains of C3 and C4 SPCH showed high polymorphism (**Figure 5**) indicating the possible functional diversity of the SPCH homologs in initiating stomatal lineage cell development.

### Crosslinks of gene expression, stomatal development and drought tolerance

**Figure 6:**
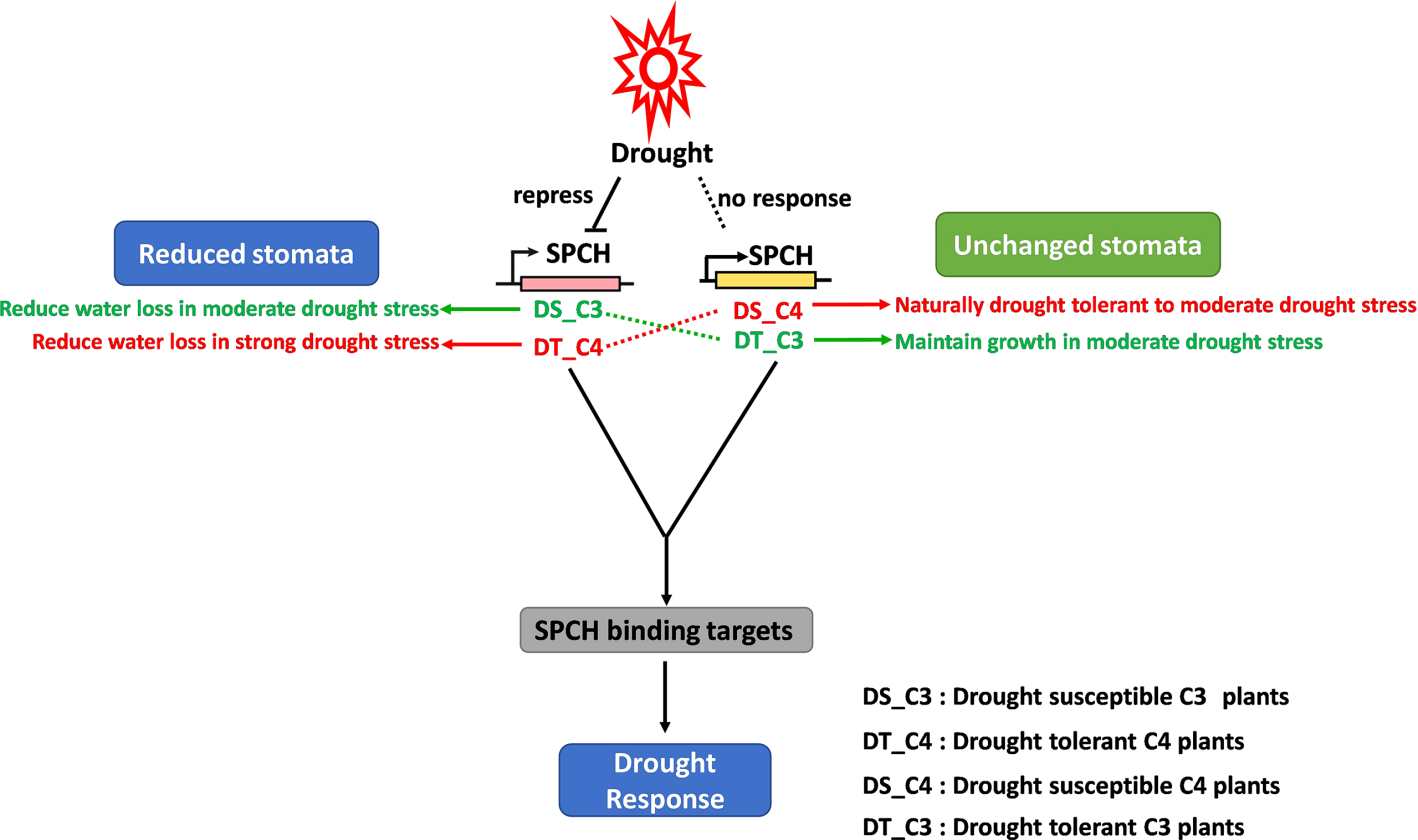
The proposed stomatal response to drought stress regulated by the divergent expression of SPCH in C3 and C4 plants. Drought repressed the expression of the SPCH of C3 plants but had no effect on that of C4 plants, resulting in the reduction of stomata in C3 plants, and no change in stomata in C4 plants in response to drought stress. However, drought-tolerant C3 plants showed no stomatal response to drought due to the stabilized expression of SPCH under drought stress. Conversely, drought-tolerant C4 plants were able to reduce the production of stomata and limit the water loss due to the re-activation of SPCH response to drought.

The relationship between gene regulatory networks of stomatal plasticity and osmotic stresses has been largely studied in C3 model plants and crops. For example, the alternative expression of SPCH *via* transcriptional and post-translational regulation directly affects stomatal production (Lampard et al., 2008; Lau et al., 2014; Lau et al., 2018), resulting in a change in drought tolerance (Hepworth et al., 2015; Yang et al., 2022). In this study, we scaled up the investigation of this relationship to C3 crops grown in tropical areas where drought stress is a big challenge for food security (Oliveira et al., 2021). We validated that the existing gene regulatory networks also worked in tropical crops (**Figures 2A–C**, **4C**). Importantly, the crosslinks of SPCH expression, stomatal production, and drought response were different in C4 crops (**Figure 6**). The homologs of *SPCH* in C4 plants have undergone a complex evolution (**Figure 3**) and showed divergent expression responses to drought stress (**Figures 4A, B**).

In conclusion, we identified the molecular difference of C3 and C4 *SPCH* at transcriptional level and protein level. We proposed a working model including the drought response of both drought-susceptible and drought-tolerant C3 and C4 crops (**Figure 6**). Our model supports the studies in C3 plants that drought represses the stomatal development *via* inhibiting the *SPCH* expression whereas the stomatal development and *SPCH* expression level are not affected in drought-tolerant C3 crops (Aharoni et al., 2004; Quan et al., 2010). In contrast, the expression of C4 *SPCH* was not affected by drought stress, which might lead to the stabilization of stomatal development of C4 plants during drought stress (**Figure 6**). However, drought-tolerant sugarcane exhibited C3 plant-like response of *SPCH* expression and stomatal development (**Figure 6**), suggesting the different upstream regulation of *SPCH* expression in drought-tolerant and drought-susceptible C4 plants. According to this result, the drought-tolerant sugarcane was able to reduce water loss *via* regulation of stomatal development to further increase the tolerance to drought stress. It would be interesting to investigate the underlying genomic and epigenetic mechanism of how *SPCH* is differentially regulated in drought-susceptible and tolerant crops. This research is also helpful in understanding the evolution of the key functional genes and their roles in drought tolerance.

## Data availability statement

The original contributions presented in the study are included in the article/**Supplementary Materials**, further inquiries can be directed to the corresponding author/s.

## Author contributions

GY conceived and supervised the project. ZS designed the study. ZS, LW and ML conducted the experiments. ZS and LW analyzed the data. ZS and GY prepared the manuscript. All authors contributed to the article and approved the submitted version.
