# Supplementary material for: The evolution and expression of stomatal regulators in C3 and C4 crops: Implications on the divergent drought tolerance
Source: Front Plant Sci. 2023 Feb 1;14:1100838. doi: 10.3389/fpls.2023.1100838 (PMC9929459; doi:10.3389/fpls.2023.1100838)
Supplement: Supplementary file 1 [file DataSheet_1.docx]

**Supplementary Table S1** The genes and corelated protein ID used for phytogenic study

| **Species** | **Gene Name** | **Protein ID** |
| --- | --- | --- |
| Arabidopsis | AtSPCH | BAB09783.1 |
|  | AtMUTE | OAP03215.1 |
|  | AtFAMA | OAP05472.1 |
|  | AtFT | NP_001320342.1 |
|  |  |  |
| Elaeis guineensis | EgSPCH | XP_010915684.1 |
|  | EgMUTE | XP_010931955.1 |
|  | EgFAMA | XP_010909228.1 |
|  | EgFT | XP_019705101.1 |
|  |  |  |
| Phoenix dactylifera | PdSPCH | XP_008783959.1 |
|  | PdMUTE | XP_008793693.1 |
|  | PdFAMA | XP_017702537.2 |
|  | PdFT | XP_038985085.1 |
|  |  |  |
| Oryza sativa.japonica | Os.J SPCH | XP_015624375.2 |
|  | Os.J MUTE | XP_015638702.1 |
|  | Os.J FAMA | XP_015638786.1 |
|  | Os.J FT | XP_015611907.1 |
|  |  |  |
| Gycine max | GmSPCH1 | XP_003523389.1 |
|  | GmSPCH2 | KAH1255720.1 |
|  | GmMUTE | KAH1217627.1 |
|  | GmFAMA | KAH1265519.1 |
|  | GmFT | BAU20339.1 |
|  |  |  |
| Triticum urartu | TuSPCH | EMS52303.1 |
|  | TuMUTE | XP_037488784.1 |
|  | TuFAMA | EMS60147.1 |
|  | TuFT | EMS47085.1 |
|  |  |  |
| Solanum lycopersicum | SlSPCH | XP_004234277.1 |
|  | SlMUTE | XP_004229565.1 |
|  | SlFAMA | XP_019071015.1 |
|  | SlFT | XP_004250075.1 |
|  |  |  |
| Solanum tuberosum | StSPCH | XP_006350638.1 |
|  | StMUTE | XP_006339401.1 |
|  | StFAMA | XP_015170256.1 |
|  | StFT | 102577452 |
|  |  |  |
| Vitis vinifera | VvSPCH | XP_002267745.1 |
|  | VvMUTE | AKA58676.1 |
|  | VvFAMA | AKA58666.1 |
|  | VvFT | 100232994 |
|  |  |  |
| Zea mays | ZmSPCH1 | XP_008659639.1 |
|  | ZmSPCH2 | PWZ23843.1 |
|  | ZmSPCH3 | PWZ07908.1 |
|  | ZmMUTE1 | XP_008656121.1 |
|  | ZmMUTE2 | NP_001106257.1 |
|  | ZmFAMA1 | PWZ08461.1 |
|  | ZmFAMA2-1 | XP_035817612.1 |
|  | ZmFAMA2-2 | XP_008656088.1 |
|  | ZmFAMA2-3 | XP_008656087.1 |
|  | ZmFT1 | PWZ16041.1 |
|  | ZmFT2 | XP_008652921.1 |
|  | ZmFT3 | XP_008671372.1 |
|  | ZmFT4 | ONM30554.1 |
|  | ZmFT5 | AQK89896.1 |
|  |  |  |
| Sorghum bicolor | SbSPCH1 | PWZ07908.1 |
|  | SbSPCH2 | XP_002437061.1 |
|  | SbMUTE1 | XP_021304028.1 |
|  | SbMUTE2 | KAG0519580.1 |
|  | SbFAMA1 | XP_002441598.2 |
|  | SbFAMA2 | XP_021303203.1 |
|  | SbFT1 | XP_002446704.1 |
|  | SbFT2 | XP_021306050.1 |
|  | SbFT3 | XP_002443085.1 |
|  | SbFT4 | XP_002457494.1 |
|  |  |  |
| Saccharum spontaneum L. | SsSPCH1-1 | Sspon.08G0008050-1A |
|  | SsSPCH1-2 | Sspon.08G0008050-3D |
|  | SsSPCH1-3 | Sspon.08G0008050-2B |
|  | SsSPCH1-4 | Sspon.08G0008050-1P |
|  | SsSPCH2-1 | Sspon.04G0026920-2C |
|  | SsSPCH2-2 | Sspon.04G0026920-1B |
|  | SsMUTE2-1 | Sspon.04G0005900-1A |
|  | SsMUTE2-2 | Sspon.04G0005900-2B |
|  | SsMUTE2-3 | Sspon.04G0005900-1T |
|  | SsMUTE3-1 | Sspon.07G0021180-2D |
|  | SsMUTE3-2 | Sspon.07G0021180-1B |
|  | SsFAMA1-1 | Sspon.01G0004830-4D |
|  | SsFAMA1-2 | Sspon.01G0004830-2B |
|  | SsFAMA1-3 | Sspon.01G0004830-1A |
|  | SsFAMA1-4 | Sspon.01G0058740-1D |
|  | SsFAMA3-1 | Sspon.07G0021180-1B |
|  | SsFAMA3-2 | Sspon.07G0021180-2D |
|  | SsFT1-1 | >Sspon.07G0037830-1T |
|  | SsFT1-2 | >Sspon.07G0037830-1D |
|  | SsFT2 | Sspon.05G0015790-1A |
|  | SsFT3-1 | Sspon.04G0008780-4D |
|  | SsFT3-2 | Sspon.04G0008780-1A |
|  | SsFT3-3 | Sspon.04G0008780-1P |
|  | SsFT3-4 | Sspon.04G0008780-3C |
|  | SsFT4 | Sspon.03G0024120-2B |

**Supplementary Table S2** Primers used in this study

| **Name** | **Sequence (5'-3')** | **Usage** |
| --- | --- | --- |
| Ss_TUBLINqrt-F | CCTACCCGAGGATCCACTTC | Q-PCR |
| Ss_TUBLINqrt-R | AAAGGCGCTGTTGGTGATTT | Q-PCR |
| Ss_SPCHqrt1-1-F | CTATGCCCGCATTTGCTCTT | Q-PCR |
| Ss_SPCHqrt1-1-R | TAGGCCACAGCATACGAGAG | Q-PCR |
| Ss_SPCHqrt1-4-F | AAGAGCAAATGCGGGCATAG | Q-PCR |
| Ss_SPCHqrt1-4-R | CTCTCGTATGCTGTGGCCTA | Q-PCR |
| Ss_SPCHqrt2-1-F | GCCATCCACAACCTTCTTCC | Q-PCR |
| Ss_SPCHqrt2-1-R | TCCTGACTTCATCCAAGCGT | Q-PCR |
| Ss_MUTEqrt-F | CTGTCCTCCGGATCCTCATC | Q-PCR |
| Ss_MUTEqrt-R | TCCTTGATCATCGTCGTCGT | Q-PCR |
| Ss_FAMAqrt-F | GTGTCGTTCGACAAGCTCAG | Q-PCR |
| Ss_FAMAqrt-R | GGCAGAGACTGGAACCTGAA | Q-PCR |
| Sb_TUBLINqrt-F | CCTACCCGAGGATCCACTTC | Q-PCR |
| Sb_TUBLINqrt-R | CACACTTGGCCATCATGGAG | Q-PCR |
| Sb_SPCHqrt1-F | AACCGGAGGAAGCAGATGAA | Q-PCR |
| Sb_SPCHqrt1-R | ACCTGCTGAAGCTCCTTGAT | Q-PCR |
| Sb_SPCHqrt2-F | TTCATCTGCTTCCTCCGGTT | Q-PCR |
| Sb_SPCHqrt2-R | ATCAAGGAGCTTCAGCAGGT | Q-PCR |
| Sb_MUTEqrt-F | CTGTCCTCCGGATCCTCATC | Q-PCR |
| Sb_MUTEqrt-R | TCCTTGATCATCGTCGTCGT | Q-PCR |
| Sb_FAMAqrt-F | TGTGAACCTGCCTAGCTACC | Q-PCR |
| Sb_FAMAqrt-R | ATGATCAGGTCGAGGAGCAG | Q-PCR |
| Os_TUBLINqrt-F | GGTTTGTCGACAGATGTGCT | Q-PCR |
| Os_TUBLINqrt-R | GACAATGGACGCTAATCGCA | Q-PCR |
| Os_SPCHqrt-F | AACCGGAGGAAGCAGATGAA | Q-PCR |
| Os_SPCHqrt-R | AGTGCAACACACACAGTGAC | Q-PCR |
| Os_MUTEqrt-F | CATCTCGAGGTGCTCCATCT | Q-PCR |
| Os_MUTEqrt-R | CCGATCTTGAGGACGAAGGA | Q-PCR |
| Os_FAMAqrt-F | GACAAGCTCACCTTCTCCGA | Q-PCR |
| Os_FAMAqrt-R | CGACTGGAACCTGAGGAAGT | Q-PCR |


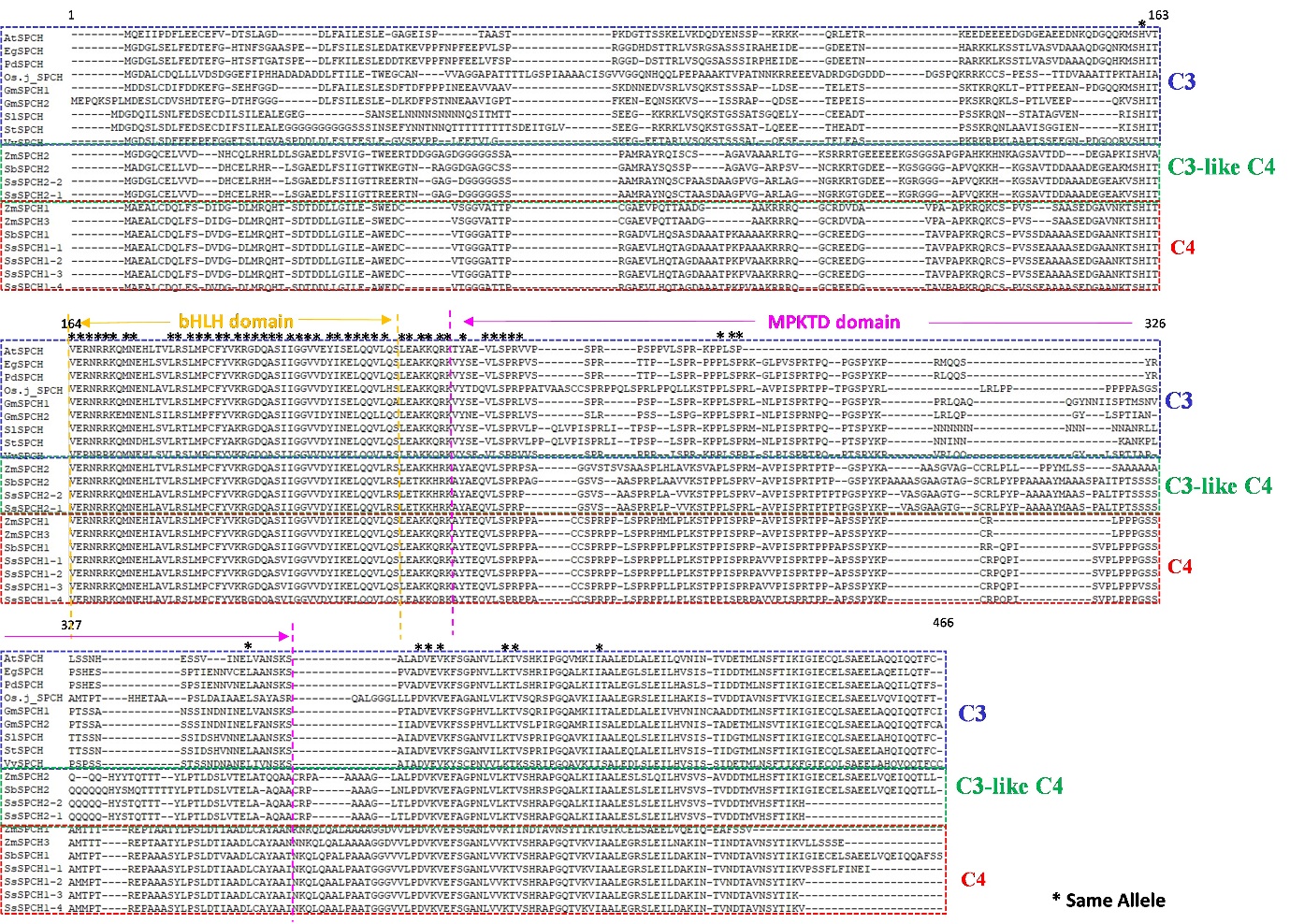


**Supplemental Figure S1** The alignment of full length SPCH of C3 and C4 plants. The blue, red and green frame indicate the bHLH sequence of C3, C4 and C3-like C4 SPCH. The arterisks indicated the same alleles.
